# The Diagnosis of Abdominal Diseases

**Published:** 1907-01-12

**Authors:** William Turner

**Affiliations:** Surgeon to the Seamen's Hospital, Greenwich


### THE DIAGNOSIS OF ABDOMINAL DISEASES.

### Being Notes of a Demonstration showing its Difficulty.

By WILLIAM TURNER, F.R.C.S.(Eng.), Surgeon to the Seamen's Hospital, Greenwich.

THE practitioners attending the London School of Clinical Medicine had before them on Monday afternoon an unusual series of cases exemplifying the difficulties of diagnosing various abdominal conditions. They had the further advantage of seeing how by careful, patient, and intelligent investigation the difficulties can be reduced or abolished.

### ENTERIC FEVER.

One case was that of a young man aged 18, complaining of general diffused pain over the abdomen, which had lasted for 10 days. There was no indication of general peritonitis, the pulse was only 60, temperature 98°, and the pain was referred generally to the umbilicus. There was no localised muscular rigidity or distension and only some tenderness on deep palpation. Examination per rectum revealed nothing abnormal. Mr. Turner stated that occasionally one sees a case with pus free in the abdomen, yet with local symptoms, and occasionally the opposite is obtained—acute severe pain, distension of the abdomen, marked rigidity, and dullness in the flanks; yet, on operating, one finds merely the appendix inflamed and no general peritonitis present. These anomalous cases are usually under the influence of morphia at the time. In this case Mr. Turner thought that the patient had no urgent symptoms requiring surgical interference, yet still it was not easy to say what was going on; the patient had just been admitted to the hospital, and the case was one for careful watching, as a lad at his age does not get continuous pain for four or five days in the abdomen for no reason. A later examination demonstrated that the patient was suffering from enteric fever. The temperature went up and he had a typical enteric stool. The pulse kept slow, but of good volume. He was removed to the enteric ward.

### LUMBAR ABSCESS.

Another patient complained of pain in the right side for six or nine months. He had no previous illness or family history of tubercle, and his age was 23. The heart was normal, the urine 1030, acid, free from albumin and blood. He was a well-developed strong-looking man, not like a patient in whom one would expect to find a movable kidney. The pain was all on the right side, and not on the left, and came on in attacks passing from the loin to the inner side of the thigh and drawing up the right testicle. On examining the abdomen, Mr. Turner was able to demonstrate a swelling on the left side occupying a large area, and not unlike an enlarged spleen. The patient had been abroad and had suffered from Malta fever. In the male three organs have to be considered in connection with such a swelling: first spleen, second kidney, third colon, and to these must be added in the female an ovarian cyst with a long pedicle. Mr. Turner next directed attention to the presence of fluctuation, which was well marked, and added that this case afforded an illustration of what specialists in abdominal diseases are wont to teach, that the patient may have symptoms on one side of the abdomen, while the diseased condition may be on the other. In this case, although the pain was right-sided, the swelling was on the left. The diagnosis of such a case is not at all easy. The condition may be caused by a cystic swelling of the left kidney—hydronephrosis, pyonephrosis, or cystic tumour. An abscess in the perirenal tissue also gives rise to similar physical signs, and in such circumstances the swelling should not move on respiration. This was then found to be the fact; and, on examining the spine, angular curvature was seen in the lower dorsal region. On questioning the patient again he stated that he had never had any injury, but he complained of jarring and pain on walking, thus confirming the diagnosis of tuberculous disease of the spine and that the swelling of the abdomen was a large lumbar abscess in connection with it. Mr. Turner pointed out that this case indicated the importance of examining a patient and trying to settle the diagnosis apart from the history. He added that the case had been mistaken for one of stone in the kidney owing to the so-called "renal pain" coming on in attacks, but the $x$-rays had failed to confirm this view. When the operation was performed an enormous abscess was emptied. The abscess extended down to the iliac fossa and had the kidney in front of it at its upper part. It was thickly encapsuled in the so-called pyogenic membrane.

Hydatids of Liver.

Mr. Turner also had a case of a woman aged 43 and suffering from a large swelling in the left epigastrium. The swelling moved with respiration, and extended beyond the middle line for about three inches. It was tense, fluctuation could be felt in it, and the patient had a history of a previous operation for hydatid of the liver at King's College Hospital about ten years ago. The conditions commenced 15 years ago, when she was residing in Buckinghamshire. The cyst was opened by an incision in the left epigastric region. It was found to be very extensive, having a diameter of between eight and ten inches. There were no less than 16 generations or colonies in the cyst, and there were no adhesions to the anterior abdominal wall. The latter fact indicates the caution which should be used in aspirating a hydatid cyst either for purposes of diagnosis or cure.

Suprapubic Operation.

Another case presenting features of interest in examination was that of a man 71 years of age, and in poor condition, who had suffered from retention of urine. He had been able to get about with difficulty until quite recently, when the pain after passing water became more decided. The urine was normal, containing no albumen, pus, or blood. Mr. Turner suspected a large prostate, and possibly stone. He made a careful rectal examination and discovered tenderness at the base of the bladder just over the prostate. On passing a silver catheter a stone could be felt at the base of the bladder. An *x*-ray photograph was taken and also revealed the presence of a large stone. The suprapubic operation was undertaken for its removal by means of which about three-quarters of an hour was saved over lithotrity, and it enabled the prostate to be dealt with if necessary. In a case like this Mr. Turner said that the bladder should be brought up to the skin, so that the great danger of the urine infiltrating the pelvic cellular tissue and setting up cellulitis is thereby diminished. The pain at the end of the penis, the absence of hæmaturia, and the frequency of micturition made him suspect stone. When hæmaturia is marked, malignant disease is the more likely diagnosis in a man of this age, but the microscopical examination of the urine will assist in the differentiation of the two.

